# Separation of Polyphenols and Caffeine from the Acetone Extract of Fermented Tea Leaves (*Camellia sinensis*) Using High-Performance Countercurrent Chromatography

**DOI:** 10.3390/molecules200713216

**Published:** 2015-07-21

**Authors:** Soo Jung Choi, Yong Deog Hong, Bumjin Lee, Jun Seong Park, Hyun Woo Jeong, Wan Gi Kim, Song Seok Shin, Kee Dong Yoon

**Affiliations:** 1 College of Pharmacy, Integrated Research Institute of Pharmaceutical Sciences, The Catholic University of Korea, Bucheon 420-743, Korea; E-Mail: wjd6694@naver.com; 2 AmorePacific R&D Unit, 314-1 Bora-dong, Giheung-gu, Yongin-si, Gyeonggi-do 449-729, Korea; E-Mails: hydhong@amorepacific.com (Y.D.H.); bjlee@amorepacific.com (B.L.); superbody@amorepacific.com (J.S.P.); misterjay@amorepacific.com (H.W.J.); katemina@amorepacific.com (W.G.K.); ssshin@amorepacific.com (S.S.S.)

**Keywords:** Camellia sinensis, tea-polyphenols, high-performance countercurrent chromatography

## Abstract

Leaves from *Camellia sienensis* are a popular natural source of various beverage worldwide, and contain caffeine and polyphenols derived from catechin analogues. In the current study, caffeine (CAF, **1**) and three tea polyphenols including (−)-epigallocatechin 3-*O*-gallate (EGCg, **2**), (−)-gallocatechin 3-*O*-gallate (GCg, **3**), and (−)-epicatechin 3-*O*-gallate (ECg, **4**) were isolated and purified by flow-rate gradient high-performance countercurrent chromatography (HPCCC) using a two-phase solvent system composed of *n*-hexane–ethyl acetate–methanol–water (1:9:1:9, *v/v*). Two hundred milligrams of acetone-soluble extract from fermented *C. sinensis* leaves was separated by HPCCC to give **1** (25.4 mg), **2** (16.3 mg), **3** (11.1 mg) and **4** (4.4 mg) with purities over 98%. The structures of **1**–**4** were elucidated by QTOF-MS, as well as ^1^H- and ^13^C-NMR, and the obtained data were compared to the previously reported values.

## 1. Introduction

**Figure 1:**
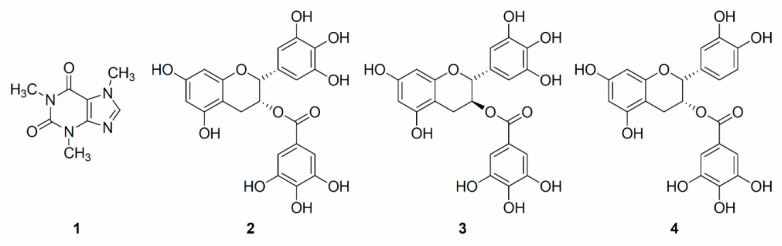
Structures of **1**–**4** from the acetone-soluble extract of fermented *C. sinensis* leaves. Compounds **1**: caffeine, **2**: (−)-epigallocatechin 3-*O*-gallate, **3**: (−)-gallocatechin 3-*O*-gallate, and **4**: (−)-epicatechin 3-*O*-gallate.

Leaves of *Camellia sinensis* are one of the most popular natural source for beverages in the tea industry worldwide [1]. It is well known that catechins-derived tea polyphenols, such as (−)-epigallocatechin 3-*O*-gallate (EGCg), (−)-gallocatechin 3-*O*-gallate (GCg), (−)-epicatechin 3-*O*-gallate (ECg), and (+)-catechin 3-*O*-gallate (Cg), are the main constituents of *C. sinensis* leaves and they show numerous beneficial functions, including anti-oxidative [2], anti-mutagenic [3], anti-inflammatory [4,5,6], and anti-obesity activities [7,8] among others. Therefore, tea polyphenols have received much attention for their potential benefits to human health, and there are many research works to isolate and elucidate the bioactive tea polyphenols. Recently, countercurrent chromatography (CCC) has been used to isolate and purify tea polyphenols [9,10,11,12,13]. CCC is a liquid-liquid chromatography which uses liquids as both the mobile and stationary phases, and it avoids chemical degradation of target compound, irreversible adsorption onto stationary phase, and sample loss during isolation process [14]. For the aforementioned reasons, CCC methods such as high-speed CCC (HSCCC) and centrifugal partition chromatography (CPC) are widely used in the natural products science field [15,16,17]. Among CCC instruments, high-performance CCC (HPCCC) is invented to retain larger amounts of stationary phase against higher mobile phase flow-rates than that of conventional CCC, because the HPCCC generates high *g*-levels (up to 240× *g*) under high rotational speed [18]. Thus, HPCCC can yield high-resolution separation with shorter separation time than conventional CCC [19]. In the current study, HPCCC using semi-preparative coil was applied to isolate and purify caffeine, (−)-epigallocatechin 3-*O*-gallate, (−)-gallocatechin 3-*O*-gallate, and (−)-epicatechin 3-*O*-gallate (Figure 1) from the acetone-soluble extract of fermented *C. sinensis* leaves (AEFC).

## 2. Results and Discussion

### 2.1. HPLC-PDA Analysis of Sample Extract

**Figure 2:**
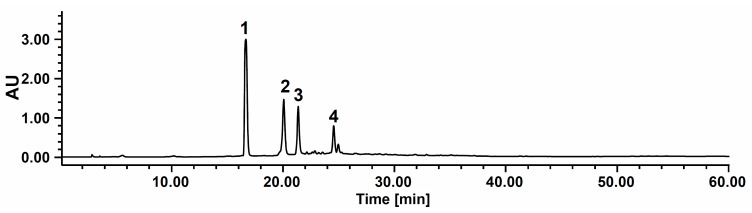
HPLC chromatogram of the acetone-soluble extract of fermented *C. sinensis* leaves. Peaks **1**: caffeine, **2**: (−)-epigallocatechin 3-*O*-gallate, **3**: (−)-gallocatechin 3-*O*-gallate, and **4**: (−)-epicatechin 3-*O*-gallate.

It is well known that commercially available tea products possess a variety of polyphenols, including (+)-catechin, (−)-epicatechin, and their derivatives, as well as caffeine [1]. In this study, AEFC was analyzed by HPLC-PDA. As shown in Figure 2, AEFC contained four main constituents including caffeine (**1**, 16.7 min), (−)-eigallocatechin 3-*O*-gallate (**2**, 19.8 min), (−)-gallocatechin 3-*O*-gallate (**3**, 21.5 min) and (−)-epicatechin 3-*O*-gallate (**4**, 24.4 min). Similar results were obtained in previouse studies, which revealed the main constituents of oolong tea (semi-fermented tea) to be CAF, EGCg, GCg, ECg and Cg [(+)-catechin 3-*O*-gallate] [7]. Based on the HPLC analysis, HPCCC experiment were performed to isolate compounds **1**–**4** as target compounds from the AEFC.

### 2.2. Evaluation of Partition Coefficient (K) Value

In conventional CCC experiments, the desirable *K* value of target compound should be in the following range: 0.2 < *K* < 2.0 [14,20]. Target molecules with *K* values less than 0.2 elute too fast to be separated, whereas compounds with *K* values greater than 2.0 exhibit better resolution between compound peaks, but yield peak dilution and long retention times under mobile phase flow-rates of 1.5–2.0 mL/min. In the current study, the *K* values of **1**–**4** were evaluated using three *n*-hexane–ethyl acetate–methanol–water (HEMWat) systems (3:7:3:7, 2:8:2:8, and 1:9:1:9, *v/v*). As shown in Table 1, the *K* values of **1**–**4** in the HEMWat 3:7:3:7 (*v/v*) system were less than 0.27, and **1** showed the largest *K* value even though its retention time (16.7 min) was the shortest (Figure 2). The *K* values of **1**–**4** in 2:8:2:8 (*v/v*) system were slightly greater than those in 3:7:3:7 (*v/v*) system, and ranged from 0.23 to 0.59, which indicated the fast, yet unresolved peak elution. In the 1:9:1:9 (*v/v*) HEMWat system, the *K* values of **1**–**4** increased dramatically to 0.61 < *K* < 3.74.

The separation factor (α; α = *K_n+1_/K_n_*) is also a significant contributor for successful CCC separation, and should be greater than 1.5 [14,20]. Separation factors between peaks **1** and **2**, **2** and **3**, and **3** and **4** were 2.28, 1.78, and 1.52, respectively, indicating that good resolution was achieved. Thus, the 1:9:1:9 (*v/v*) HEMWat system was selected as the suitable two-phase solvent system for the semi-preparative HPCCC of AEFC.

**Table 1:** Partition coefficient values (*K*) of compounds **1**–**4** in *n*-hexane–ethyl acetate–methanol–water (HEMWat) systems. Peaks **1**: caffeine, **2**: (−)-epigallocatechin 3-*O*-gallate, **3**: (−)-gallocatechin 3-*O*-gallate, and **4**: (−)-epicatechin 3-*O*-gallate. (α: separation factor; α_1_ *= K*_2_*/K*_1_, α_2_ *= K*_3_*/K*_2_, α_3_ *= K*_4_*/K*_3_).

HEMWat System (*v/v*)	Partition Coefficient (*K*) Value						
	1 (*K*_1_)	α_1_	2 (*K*_2_)	α_2_	3 (*K*_3_)	α_3_	4 (*K*_4_)
3:7:3:7	0.27	-	0.03	2.33	0.07	2.14	0.15
2:8:2:8	0.39	-	0.23	2.04	0.47	1.74	0.82
1:9:1:9	0.60	2.28	1.37	1.78	2.45	1.52	3.74

### 2.3. HPCCC Separation of Sample Extract

**Figure 3:**
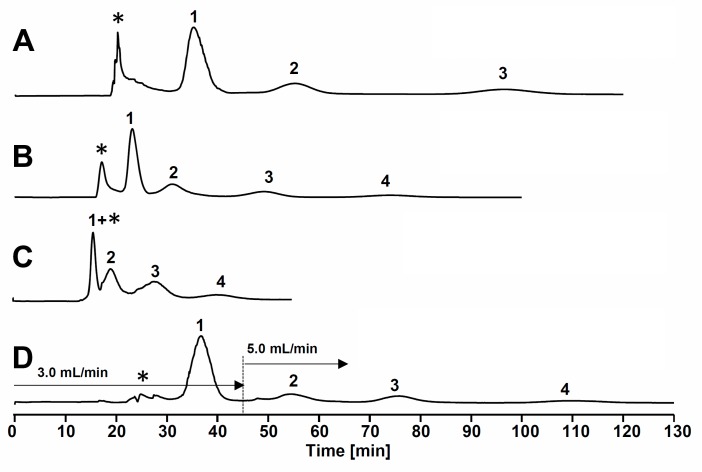
HPCCC separation patterns of the acetone-soluble extract of fermented *C. sinensis* leaves using *n*-hexane–ethyl acetate–methanol–water (1:9:1:9, *v/v*) system with rotational speed at 1600 rpm. (**A**) flow-rate at 3.0 mL/min; (**B**) flow-rate at 5.0 mL/min; (**C**) flow-rate at 8.0 mL/min; (**D**) gradient flow-rate at 3.0 mL/min in 0–45 min, and 5.0 mL/min in 45–130 min. Peaks **1**: caffeine, **2**: (−)-epigallocatechin 3-*O*-gallate, **3**: (−)-gallocatechin 3-*O*-gallate, and **4**: (−)-epicatechin 3-*O*-gallate.

Figure 3 shows the actual semi-preparative HPCCC patterns of 20 mg of AEFC using the HEMWat (1:9:1:9, *v/v*) system, with a rotational speed at 1600 rpm and different mobile phase flow-rates. A flow-rate at 3.0 mL/min gave the best resolution, but the elution time of **4** was greater than 130 min (Figure 3A). The elution time was shortened by increasing the flow-rate to 5.0 mL/min, but **1** was slightly contaminated by a nearby polar impurity (*), and the peak resolution between **1** and **2** was worse than that of 3.0 mL/min condition (Figure 3B). A flow-rate at 8.0 mL/min resulted in the fast elution of compounds **1**–**4**, but **1** co-eluted with a polar impurity (*) and an unsatisfactory separation between compound peaks was observed (Figure 3C). Finally, a flow-rate gradient method (3.0 mL/min in 0–45 min and 5.0 mL/min in 45–130 min) was successfully applied for the optimal separation of **1**–**4**, and good peak resolutions were obtained (Figure 3D).

**Figure 4:**
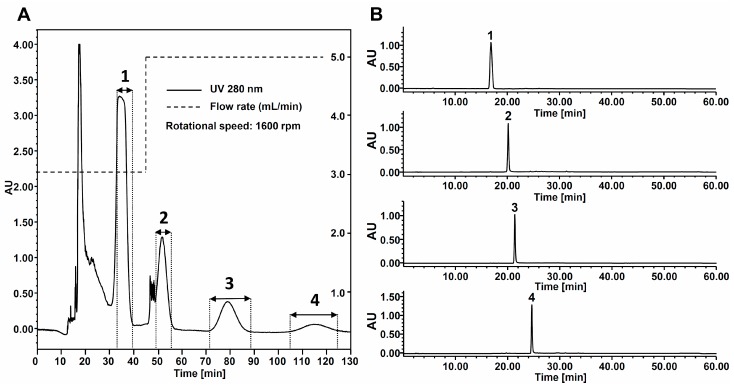
(**A**) Preparative HPCCC separation of the acetone-soluble extract of fermented *C. sinensis* leaves using an *n*-hexane–ethyl acetate–methanol–water (1:9:1:9, *v/v*) system. (**B**) HPLC chromatograms of compound **1**–**4** isolated by HPCCC. Peaks **1**: caffeine, **2**: (−)-epigallocatechin 3-*O*-gallate, **3**: (−)-gallocatechin 3-*O*-gallate, and **4**: (−)-epicatechin 3-*O*-gallate.

Under the aforementioned gradient flow-rate conditions, 200 mg of AEFC was separated by HPCCC using semi-preparative coil to give **1** (25.4 mg), **2** (16.3 mg), **3** (11.1 mg) and **4** (4.4 mg) with purities over 98% (Figure 4) and the structures of **1**–**4** were confirmed by QTOF-MS, ^1^H- and ^13^C-NMR; the obtained data were compared to previously reported values. The stationary phase retention was 67.8% after the HPCCC experiment, which indicated the high retention of stationary phase in high flow-rate condition.

Thus far, several research groups have reported the use of CCC for the separation of tea-catechin related polyphenols. Yanagida *et al.* used analytical HSCCC to separate tea-polyphenols from commercially available tea leaves using a *tert*-butyl methyl ether–acetonitrile–0.1% aqueous trifluoroacetic acid (2:2:3, *v/v*, normal phase mode, flow-rate: 2 mL/min) [9]. Regarding the semi-preparative or preparative CCC separation of tea-polyphenols, Wang *et al.* described the semi-preparative HSCCC separation of EGC, EGCg, GCg and ECg from 30 mg of green tea using an *n*-hexane–ethyl acetate–methanol–water–acetic acid system [1:5:1:5:0.25 (*v/v*); reversed-phase mode; flow-rate: 2 mL/min; rotational speed: 800 rpm] in 200 min, but further recrystallization and decaffeination were used to yield pure tea-polyphenols after CCC separation [11]. Industrial-scale low-speed rotary countercurrent chromatography (LRCCC) was used to yield 40.05 g of EGCg (purity 92.7%) from 150 g of dried extract of tea leaves. The EGCg isolation was achieved by using a two-phase solvent system composed of *n*-hexane–ethyl acetate–1-butanol–water–acetic acid [0.5:1:2:6:0.2 (*v/v*); reversed-phase mode; flow-rate: 5 mL/min; rotational speed: 21 rpm), and the run time was 72 h [21].

It is hard to compare absolutely the current HPCCC study with such previous CCC results, because the separation scale and suitable two-phase solvent systems were different. However, rapid one-step isolation and purification of pure CAF, EGCg, GCg, and ECg from 200 mg of AEFC could be accomplished in this study using a flow-rate gradient semi-preparative HPCCC by (1) appropriate selection of two-phase solvent system to obtain the desired *K* values for better CCC peak resolution and more pure compounds than those in previous literature, and (2) by using a high mobile phase flow-rate to shorten the CCC run time (130 min). The present study provides useful information regarding the separation of tea-polyphenols using HPCCC.

*Caffeine* (**1**): positive-ion QTOF-MS: *m/z* 195.0870 [M + H]^+^ [calcd 195.0882 for C_8_H_11_N_4_O_2_); ^1^H-NMR (500 MHz, CD_3_OD): 8.00 (1H, s, H-8), 3.87 (3H, s, H-12), 3.40 (3H, s, H-11), 3.21 (3H, s, H-10); ^13^C-NMR (125 MHz, CD_3_OD): 154.4 (C-6), 151.1 (C-2), 148.1 (C-4), 142.8 (C-8), 106.6 (C-5), 33.1 (C-12), 29.4 (C-11), 27.5 (C-10). The spectroscopic data obtained were compared to the previously reported values [22], and the compound was identified as caffeine.

*(−)-Epigallocatechin 3-O-gallate* (**2**): positive-ion QTOF-MS: *m/z* 459.0912 [M + H]^+^ [calcd 459.0927 for C_22_H_19_O_11_); ^1^H-NMR (500 MHz, CD_3_OD): 6.95 (2H, s, H-2′′,6′′), 6.50 (2H, s, H-2′,6′), 5.96 (2H, s, H-6,8), 5.53 (1H, m, H-3), 4.97 (1H, s, H-2), 2.98 (1H, dd, *J* = 17.3, 4.6 Hz, H-4a), 2.84 (1H, dd, *J* = 17.4, 2.2 Hz, H-4b); ^13^C-NMR (125 MHz, CD_3_OD): 167.8 (COO), 158.0 (C-7), 158.0 (C-5), 157.4 (C-9), 146.8 (C-3′,5′), 146.4 (C-3′′,5′′), 139.9 (C-4′′), 133.9 (C-4′), 131.0 (C-1′), 121.7 (C-1′′), 110.4 (C-2′′,6′′), 107.0 (C-2′,6′), 99.6 (C-10), 96.7 (C-6), 96.0 (C-8), 78.7 (C-2), 70.1 (C-3), 27.0 (C-4). The spectroscopic data obtained were compared to the previously reported values [23], and the compound was elucidated as (−)-epigallocatechin 3-*O*-gallate.

*(−)-Gallocatechin 3-O-gallate* (**3**): positive-ion QTOF-MS: *m/z* 459.0914 [M + H]^+^ [calcd 459.0927 for C_22_H_19_O_11_); ^1^H-NMR (500 MHz, CD_3_OD) : 6.97 (2H, s, H-2′′,6′′), 6.40 (2H, s, H-2′,6′), 5.95 (2H, s, H-6,8), 5.38 (1H, q, *J* = 5.2 Hz, H-3), 5.05 (1H, d, *J* = 5.2 Hz, H-2), 2.76 (1H, dd, *J* = 16.5, 5.0 Hz, H-4a), 2.71 (1H, dd, *J* = 16.5, 5.3 Hz, H-4b); ^13^C-NMR (125 MHz, CD_3_OD): 167.8 (COO), 158.3 (C-7), 157.8 (C-5), 156.5 (C-9), 147.1 (C-3′,5′), 146.5 (C-3′′,5′′), 140.0 (C-4′′), 134.1 (C-4′), 131.2 (C-1′), 121.6 C-1′′), 110.3 (C-2′′,6′′), 106.5 (C-2′,6′), 99.7 (C-10), 96.6 (C-8), 95.8 (C-6), 79.4 (C-2), 71.2 (C-3), 23.9 (C-4). The spectroscopic data obtained were compared to the previously reported values [24], and the compound was elucidated as (−)-gallocatechin 3-*O*-gallate.

*(−)-Epicatechin 3-O-gallate* (**4**): positive-ion QTOF-MS: *m/z* 443.0959 [M + H]^+^ [calcd 443.0978 for C_22_H_19_O_10_); ^1^H-NMR (500 MHz, CD_3_OD): 6.95 (2H, s, H-2′′,6′′), 6.93 (1H, s, H-2′), 6.81 (1H, d, *J* = 8.2 Hz, H-6′), 6.69 (1H, d, *J* = 8.2 Hz, H-5′), 5.96 (2H, s, H-6,8), 5.53 (1H, s, H-3), 5.03 (1H, s, H-2), 2.99 (1H, dd, *J* = 17.3, 4.5 Hz, H-4a), 2.85 (1H, d, *J* = 17.3 Hz, H-4b); ^13^C-NMR (125 MHz, CD_3_OD): 167.8 (COO), 158.1 (C-7), 158.0 (C-9), 157.4 (C-5), 146.5 (C-3′′,5′′), 146.1 (C-3′,4′), 140.0 (C-4′′), 131.6 (C-1′), 121.6 (C-1′′), 119.5 (C-6′), 116.2 (C-5′), 115.3 (C-2′), 110.4 (C-2′′,6′′), 99.6 (C-10), 96.7 (C-6), 96.1 (C-8), 78.8 (C-2), 70.1 (C-3), 27.0 (C-4). The spectroscopic data obtained were compared to the previously reported values [25], and the compound was identified as (−)-epicatechin 3-*O*-gallate.

## 3. Experimental Section

### 3.1. General Experimental Procedures

The HPCCC used in this study was a Spectrum (Dynamic Extractions, Berkshire, UK). The Spectrum contained two sets of bobbin and each bobbin contained an analytical column (bobbin 1, 13 mL; bobbin 2, 12 mL; 0.8 mm I.D.), and a semi-preparative coil (bobbin 1, 70.5 mL; bobbin 2, 69.5 mL; 1.6 mm I.D.). The β-values of coil was ranged 0.52 to 0.86. The Spectrum was coupled to an IOTA S 300 pump (Ecom, Prague, Czech Republic), a 2487 dual λ absorbance detector (Waters, Milford, MA, USA), a Foxy R2 fraction collector (Teledyne Isco, Lincoln, NE, USA) and a CCA-1111 circulatory temperature regulator (Eyela, Tokyo, Japan) which maintained the internal HPCCC temperature at 30 °C. HPLC analyses were performed by an Alliance HPLC system (Waters, Milford, MA, USA) with an Eclipse XDB-C18 column (4.6 ´ 250 mm I.D., 5 μm, Agilent Technologies, Santa Clara, CA, USA). Structure elucidation of compounds from HPCCC peak fractions were conducted by an AVANCE 500 spectrometer (Bruker, Karlsruhe, Germany) for ^1^H- and ^13^C-NMR, a 6460 QTOF mass spectrometer (Agilent Technologies). Organic solvents (analytical grade) for HPCCC separation were obtained from Daejung-Chemical Industry Co. Ltd. (Gyeongsangbuk-do, Goryeong-gun, Korea). Ultrapure water was generated by a Millipore Milli-Q water purification system (Millipore, Billerica, MA, USA). Acetonitrile for HPLC analysis was purchased from Fisher Scientific Korea Ltd. (Seoul, Korea). The acetone-soluble extract of the fermented *C. sinensis* leaves was provided by Amorepacific Corporation R&D Unit (Seoul, Korea).

### 3.2. Preparation of Sample Extract

Fresh green tea (*C. sinensis*) leaves were collected in spring from Osulloc Tea Garden in Jeju, Korea. Tea fermentation was carried out at 50 °C for 3 days and then 80 °C for 4 days followed by inoculation of *Bacillus subtilis* spp. isolated from traditional Korean soybean paste, and incubation for 4 day at 80 °C. After completing the fermentation process, the fermented tea was dried at 80 °C for 4–5 h and stored at room temperature for 100 days. The detailed methods for producing the fermented tea used in this study are fully described in a patent with International application number, PCT/KR2009/006979. Dried fermented tea (500 g) was extracted three times with acetone (6.0 L) using ultrasonic bath and evaporated under reduced pressure to give an acetone-soluble extract (21.4 g). The acetone-soluble extract of fermented tea was stored in a refrigerator (−20 °C) prior to HPLC analysis and HPCCC experiment.

### 3.3. HPLC-PDA Analysis

The acetone-soluble extract of the fermented *C. sinensis* leaves and HPCCC peak fractions were analyzed by HPLC-PDA using a Zorbax C18 column (250 × 4.6 mm, I.D., 5 μm; Agilent Technologies). The mobile phases were acetonitrile for solvent A (acidified by 0.01% trifluoroacetic acid) and water (acidified by 0.01% trifluoroacetic acid) for solvent B. The mobile phase gradient was 5% A (0–5 min), 5%–30% A (5–30 min), 30%–100% A (30–50 min), and 100% A (50–60 min). The mobile phase flow-rate was 1.0 mL/min and the injection volume was 20 μL. The HPLC chromatogram was monitored at UV 280 nm.

### 3.4. Evaluation of Partition Coefficient Value

The partition coefficient (*K*) values of target compounds were evaluated by HPLC-PDA analysis. Three ratios of two-phase solvent systems composed of *n*-hexane–ethyl acetate–methanol–water (HEMWat, 3:7:3:7, 2:8:2:8, 1:9:1:9, *v/v*) were used in this study. Approximately 10 mg of acetone soluble extract of *C.* *sinensis* was added to a vial (20 mL) containing 1:1 mixture of the upper and lower phase (each 5 mL) of above two-phase systems. The vial was shaken vigorously to achieve equilibration of target compound between upper and lower phases. After 10 min, the upper and lower phases were separated, and 1 mL of each phase was taken in a vial (4 mL) followed by removing the solvent under N_2_ gas. The residue in a vial was dissolved in methanol and subjected to HPLC. The *K* value of each target compound was calculated as the peak area of target compound in the stationary phase (upper phase) divided by that in the mobile phase (lower phase).

### 3.5. HPCCC Procedure

In order to select the optimal HPCCC condition, actual HPCCC experiments using semi-preparative coil were performed using HEMWat (1:9:1:9, *v/v*) system and small amount of AEFC (20 mg). The semi-preparative coil was filled with upper aqueous phase with a flow-rate at 10 mL/min. After filling stationary phase in the column, the HPCCC was rotated at 1600 rpm while the lower mobile phase was pumped in head-to-tail mode at flow-rates of 3.0–8.0 mL/min. The sample extract (20 mg) was dissolved in 1:1 mixture of lower and upper phase (1 mL each) and was injected to HPCCC after the mobile phase had emerged in the effluent. The HPCCC chromatograms were monitored by UV at 280 nm. After selecting the optimal HPCCC condition, the final HPCCC using semi-preparative column was performed according to the result of above HPCCC separation patterns. The HPCCC condition was as follows; two-phase solvent system: HEMWat (1:9:1:9, *v/v*), rotational speed: 1600 rpm, flow-rate: 3.0 mL/min (0–45 min), 5.0 mL/min (45–130 min), and the amount of AEFC: 200 mg dissolved in 1:1 mixture of upper and lower phases (3 mL each). After HPCCC separation, solvents retained in the column were pushed by N_2_ gas and collected to measure the retention of stationary phase.

### 3.6. Identification of HPCCC Peak Fractions 

Structure elucidation of **1**–**4** in HPCCC peak fractions were conducted through the analysis of QTOF-MS, ^1^H- and ^13^C-NMR spectroscopic data which were compared to the previously reported values [21,22,23,24].
